# Complex Cardiovascular Morbidities in Prader-Willi Syndrome: A Multidisciplinary Approach

**DOI:** 10.7759/cureus.56591

**Published:** 2024-03-20

**Authors:** Raul Alba, Soroush Omidvarnia, Jared J Bies, Tim Carlson, Qusay Alfaori, Thwe Htay

**Affiliations:** 1 Internal Medicine, Texas Tech University Health Sciences Center El Paso, El Paso, USA; 2 Medicine, Texas Tech University Health Science Center El Paso, El Paso, USA; 3 Internal Medicine, Texas Tech University Health Science Center El Paso, El Paso, USA; 4 Medical Education, Texas Tech University Health Sciences Center El Paso, El Paso, USA

**Keywords:** eisenmenger syndrome, prader-willi syndrome, multi-disciplinary teams, cardio vascular disease, pws

## Abstract

This case emphasizes the complexity of Prader-Willi syndrome (PWS), the need for a collaborative approach from specialists, and a closer look at the various cardiovascular complexities associated with this syndrome. While current treatments focus on managing symptoms, ongoing genetic research offers hope for more favorable outcomes. Further studies are crucial to gauge the effectiveness of these treatments for PWS patients. We detail a patient with a complex medical history of PWS, further complicated by congenital heart disease with Eisenmenger’s syndrome, diabetes mellitus, pulmonary hypertension, venous insufficiency, hypothyroidism, and hyperlipidemia. Reported in this study is a compilation of clinical data as well as suggestions from several medical specialists in applying a multifaceted approach to treatment, significantly emphasizing the need for interdisciplinary care and management of patients experiencing a combination of various medical issues with an emphasis on cardiovascular complications.

## Introduction

We present a case report of a 20-year-old female with Prader-Willi Syndrome (PWS), a rare and complex genetic disorder that is the leading genetic cause of life-threatening obesity in humans [1]. PWS results from the loss of expression of paternally derived genes in the PWS critical region on chromosome 15q11-q13, which can occur due to a paternal deletion, maternal uniparental disomy 15, or an imprinting defect [2,3].

Although cardiovascular abnormalities are known to be associated with PWS, such as congenital heart defects and myocardial dysfunction [4], our patient had an unusual combination of multiple medical conditions, including Eisenmenger’s syndrome, congestive heart failure, diabetes mellitus, pulmonary hypertension, venous insufficiency, hypothyroidism, and hyperlipidemia. This case illustrates the necessity for a multidisciplinary approach in managing PWS patients with complex and co-existing medical problems and delves into the various cardiovascular complications associated with PWS.

## Case presentation

A 20-year-old female with a past medical history of PWS, secundum ASD and multiple fenestrations with predominantly right to left shunt (Eisenmenger’s syndrome), congestive heart failure, diabetes mellitus, severe pulmonary hypertension, venous insufficiency, hypothyroidism, and hyperlipidemia presented to the emergency department for worsening anasarca and respiratory distress. The patient had multiple similar presentations as a result of her worsening clinical status.

The patient’s previous medication regimen included macitentan, riociguat, and sildenafil and discontinued them due to intolerable side effects. The patient’s medications upon presentation consisted of furosemide 40 mg daily, Insulin glargine 20 units twice daily, and levothyroxine 100 mcg daily. The patient used 3L nasal cannula oxygen at a baseline. Her family history was significant for diabetes mellitus in her father, and she denied the use of tobacco, alcohol, or illicit drugs.

On physical examination, the patient presented with a temperature of 36.7 °C, a heart rate of 83 (peripheral), and an oxygen saturation of 90% with 3L of nasal canular O2. The patient was vitally stable, alert, oriented, and well-nourished. Lung bases were decreased on auscultation bilaterally, non-labored respiration was present, and 3+ pitting edema in her lower extremities. An 8-cm-wide venous stasis ulcer on the left lower limb was present. Initial laboratory findings showed a WBC count of 9.87 × 10^3^/UL, hemoglobin of 17.1 g/dL, and hematocrit of 58.2% (Table 1).

**Table 1:** Initial laboratory findings

Lab	Patient value	Reference ranges
White blood cell count (WBC)	9.87 x 10^3^/UL	4.50-11.03 x 10^3^/UL
Reb blood cell count (RBC)	5.91 x x 10^6^/UL High	3.50-5.50 x 10^6^/UL
Hemoglobin	17.1 g/dL High	12.0-15.0 g/dL
Hematocrit	58.2% High	40-50% (male), 36-46% (female)
Platelets	174 x 103/µL	150-450 x 10^3^/µL
Sodium, serum	136 mmol/L	135-145 mmol/L
Potassium, serum	4.8 mmol/L	3.5-5.0 mmol/L
Chloride, serum	104 mmol/L	98-108 mmol/L
Blood urea nitrogen, serum	27 mg/dL High	7-20 mg/dL
Creatinine	0.9 mg/dL	0.6-1.3 mg/dL
Calcium, serum	8.1 mg/dL Low	8.5-10.5 mg/dL
Magnesium, serum	1.6 mg/dL	1.7-2.2 mg/dL
Albumin, serum	3 g/dL Low	3.5-5.0 g/dL
N-terminal pro-BNP (NT-proBN)	5,290 pg/mL	<300 pg/mL
Cholesterol, serum	187 mg/dL	<200 mg/dL
Triglycerides, serum	150 mg/dL	<150 mg/dL
HDL Cholesterol, serum	25 mg/dL Low	40 (men), >50 (women) mg/dL
LDL Calculated	132 mg/dL High	<100 mg/dL

Hospital course

Respiratory Support

High-flow nasal canula was utilized to sustain adequate oxygen levels upon admission. Our patient underwent a trial of pulmonary vasodilators including sildenafil and macitentan but was unable to tolerate these treatments. Oxygenation via high-flow nasal canula was continued and gradually weaned to a nasal canula as tolerated.

Cardiovascular Management

**Figure 1:**
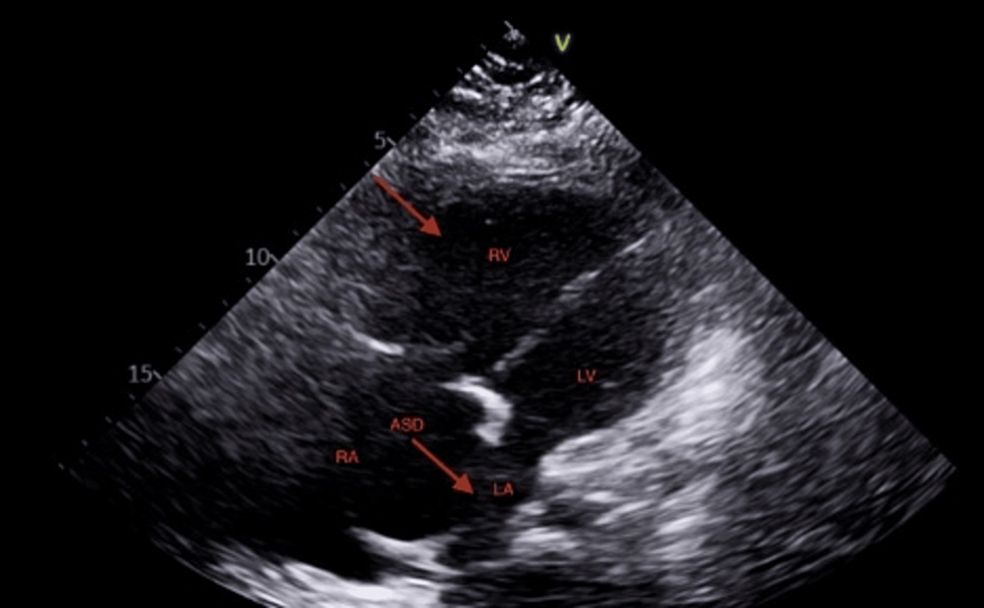
Transthoracic echocardiogram Right ventricle and atrial septal defect marked by red arrows.

Cardiovascular management involved conducting comprehensive imaging studies. A transthoracic cardiac echocardiogram showed the right atrium and right ventricle (RV) severely dilated with depressed RV systolic function. Small LV size with flattened interventricular septum consistent with RV pressure/volume overload (Figure 1). Right heart catheterization further confirmed the presence of severe right heart failure.

Renal and Metabolic Stabilization

Our patient underwent renal ultrafiltration to address pulmonary hypertension but was unable to complete the full treatment due to intolerance. Nephrotoxic medications were strictly avoided to preserve renal function, and strategies for maintaining hydration were implemented. The patient continued diuresis with IV furosemide, with closed monitoring of oxygenation, strict intake/output, electrolyte balance, daily weights, and kidney function. Hypoglycemia was managed by discontinuing her home insulin regimen and initiating insulin sliding scale for optimizing blood glucose levels.

Wound care management

The wound identified on left anterior tibia, diagnosed as a venous stasis ulcer, required specialized wound care and a multidisciplinary team approach. Patient received meticulous dressing changes, regular wound assessments, and specific wound care to expedite healing and infection prevention.

Throughout her hospitalization, our patient underwent an intricate treatment regimen comprising respiratory support, attempted vasodilator therapy, vigilant cardiovascular management, renal function preservation, glucose regulation, and specialized wound care. Our patient’s cardiovascular and respiratory status returned to baseline with 2-3L nasal cannula oxygen, and her acute kidney injury (AKI) resolved, returning to within normal limits. The patient’s wound was last assessed with no signs or symptoms of infection noted. The patient was discharged home with oral daily furosemide and continuous oxygenation, along with scheduled outpatient follow-up and wound care.

## Discussion

This case highlights the multifaceted considerations necessary for managing a patient with PWS and multiple co-occurring conditions that result from severe pulmonary hypertension and right heart failure. Particularly, a need for multi-specialty collaboration and careful monitoring to improve the patient’s overall health and well-being. The care of our patient is orchestrated by a multidisciplinary team including specialists in cardiology, pulmonology, nephrologists, internal medicine, endocrinology, nutrition, respiratory care, therapy (occupational, speech, physical), and wound care. Together, they collaboratively delivering comprehensive treatment.

PWS is a condition involving a complicated mixture of genetic factors and cardiovascular comorbidities. Numerous cases consistently showed how young adults with PWS often suffer from cardiovascular risk factors such as high cholesterol, diabetes, and high blood pressure, demonstrating cardiovascular vulnerability within the young adult demographic [5]. In particular, higher levels of C-reactive protein, which are linked to underlying inflammation, exacerbate the intricate cardiovascular risks in patients with PWS [6].

One case report detailed a 27-year-old man with PWS and multiple underlying comorbidities such as obesity, hypertension, diabetes mellitus, and dyslipidemia, which led to three-vessel coronary artery disease (CAD) [7]. These findings emphasize how patients with PWS are at greater risk of early-onset heart disease, stressing the urgent need for more specific strategies to manage cardiovascular risks.

Another study showcased a 10-year-old boy with PWS who was experiencing sinus pauses, calling attention to the importance of careful heart rate monitoring in younger individuals who suffer from PWS [8]. This patient’s subsequent need for a pacemaker status-post syncope indicates potential arrhythmia risks, further supporting the notion of ongoing heart rhythm studies for children with PWS, aiding in the prevention of serious complications in the future. Additionally, the literature explains the broader effects of obesity, both idiopathic and syndromic, on cardiovascular health [9, 10]. While idiopathic obesity and cardiovascular consequences are extensively studied, these studies accentuate the shortage of data on syndromic obesity in conditions like PWS, characterized by heterogeneous phenotypes and premature mortality, placing emphasis on the crucial need for more research to understand more specific cardiovascular risks linked to syndromic obesity in individuals with PWS [11,12].

PWS is a rare genetic disorder that affects multiple body systems and causes various clinical features throughout the patient’s life span [13]. The diagnosis of PWS is based on clinical criteria and confirmed by genetic testing [2]. The management of PWS involves addressing various health issues as they arise, such as growth hormone deficiency, hypogonadism, obesity, diabetes mellitus, sleep apnea, behavioral problems, and cardiovascular complications [14,15]. There is no cure for PWS, and the current treatments are mainly symptomatic and supportive [16]. However, recent advances in understanding the genetic mechanisms underlying PWS have opened new avenues for potential genetic and epigenetic therapies that could restore the expression of the silenced genes or modulate their downstream effects [17,18]. Further research is needed to explore the safety and efficacy of these novel approaches in PWS patients.

## Conclusions

The intricacies presented in this case highlight the complexities faced by individuals with PWS and underscore the importance of a multidisciplinary approach when managing such patients. Illustrated in this report are the dedications and resilience held by a multidisciplinary team, demonstrating how these efforts help in delivering quality healthcare. Further need for research and investigation into various treatment modalities for PWS is crucial for improving long-term outcomes, particularly in the fields of genetics and epigenetic therapies. We present this to stimulate further interest and investigation into this syndrome, ultimately leading to enhanced quality healthcare delivery for these patients.
